# Morphology-Tailored Dynamic State Transition in Active-Passive Colloidal Assemblies

**DOI:** 10.34133/research.0304

**Published:** 2024-01-24

**Authors:** Nan Yu, Zameer H. Shah, Mingcheng Yang, Yongxiang Gao

**Affiliations:** ^1^ Institute for Advanced Study, Shenzhen University, 518060, Shenzhen, China.; ^2^ Key Laboratory of Optoelectronic Device and Systems of Ministry of Education and Guangdong Province, College of Optoelectronic Engineering, Shenzhen University, 518060, Shenzhen, China.; ^3^ Beijing National Laboratory for Condensed Matter Physics and Laboratory of Soft Matter Physics, Institute of Physics, Chinese Academy of Sciences, Beijing 100190, China.; ^4^ School of Physical Sciences, University of Chinese Academy of Sciences, Beijing 100049, China.; ^5^ Songshan Lake Materials Laboratory, Dongguan, Guangdong 523808, China.

## Abstract

Mixtures of active self-propelled and passive colloidal particles promise rich assembly and dynamic states that are beyond reach via equilibrium routes. Yet, controllable transition between different dynamic states remains rare. Here, we reveal a plethora of dynamic behaviors emerging in assemblies of chemically propelled snowman-like active colloids and passive spherical particles as the particle shape, size, and composition are tuned. For example, assembles of one or more active colloids with one passive particle exhibit distinct translating or orbiting states while those composed of one active colloid with 2 passive particles display persistent “8”-like cyclic motion or hopping between circling states around one passive particle in the plane and around the waist of 2 passive ones out of the plane, controlled by the shape of the active colloid and the size of the passive particles, respectively. These morphology-tailored dynamic transitions are in excellent agreement with state diagrams predicted by mesoscale dynamics simulations. Our work discloses new dynamic states and corresponding transition strategies, which promise new applications of active systems such as micromachines with functions that are otherwise impossible.

## Introduction

Active materials composed of energy-consuming self-propelled components and passive entities are common in nature [1]. Through self-powered motion and ensuing nonequilibrium interactions, such materials display dynamic functions that are central to survival, resource acquisition, and reproduction of living organisms [2]. For example, the attachment of whip-like flagellum to rotary motor allows bacteria to navigate through a fluid [3]. By changing the configuration of flagella, bacteria can further alternate between different modes of motion, such as run and tumble [4], stop and coil [5], push and pull [6], and even wrap [7], in response to environmental cues. Molecular protein motors interacting with cytoskeletal network composed of long microtubule and actin filaments allows Eukaryotic cells to accomplish complex functions such as intracellular transportation, cell division, and motility. Cells can even switch the direction cargo transport along microtubule highway to ensure efficient delivery depending on their needs [8]. In fact, in vitro experiments have shown remarkable dynamic behaviors and self-organization in mixture of active motor proteins powered by adenosine triphosphate with passive microtubule [9] and actin filaments [10]. It has further been shown that protein motors can be programmed to transport on synthetic DNA tracks [11]. It is of substantial interest to design synthetic materials containing self-driven units to realize dynamic functions that are otherwise impossible [1].

Significant efforts have been placed on active materials that contain self-propelled colloids. For example, active colloids have been found to exhibit persistent orbiting dynamics around giant unilamellar vesicles [12]. Encapsulation of active colloids inside giant unilamellar vesicles has led to various membrane shape that may be useful for designing artificial cells [13]. Assemblies of active colloidal motors with lipid tubes have revealed kinesin/microtubule type of directional transportation of lipid vesicles [14]. Another important class of synthetic active materials are mixtures of active self-propelled colloids and passive ones [15]. Chemically propelled active colloids may attract one or multiple passive colloids via electrostatic [16], hydrophobic [17], and phoretic [18–21] interactions, self-assembling into translating clusters that hold applications in cargo loading and transportation, or exhibiting predator–prey type biomimetic functions [22,23]. Active colloids have also been observed to orbit around passive spheres [24] or fixed posts [25]. When multiple active colloids assemble with passive ones, they may also form rotating or translating clusters [19,21,26]. With the assistance of optical trapping, much more complex assemblies such as cogwheels can be assembled [27]. Yet achieving more sophisticated dynamics via the interplay of active and passive colloids, especially on tailoring transitions between different dynamic states remains rare [28], despite their great potential in designing microscopic robotics [29] and machines [30,31].

Here, we reveal rich dynamics in mixtures of chemically propelled snowman-like Janus active colloids and passive spherical colloids at room temperature in the dilute regime. We observe that their assemblies display 3 dynamic states, translational state only, orbiting state for one active particle and translational state for 2 active particles, and orbiting states only as the extruded active part of the Janus colloid is increased. The translating mode benefits the transportation of large cargoes, whose transportation speed increases with the number of active colloids. In addition, the translating mode facilitates the assembly of active colloids around a passive one into sun-flower-like structures with full occupancy determined by the size of the passive particle. In the orbiting mode, we find that the linear translational speed of colloids is sensitive to their orientational arrangement at the perimeter of the passive colloid, less sensitive to the number of active colloids, and essentially insensitive to the size of the passive colloid. Most interestingly, we observe that the assemblies of one active colloid with 2 passive ones may transit from persistent “8”-like cyclic motion to hopping between circling states around one passive particle in the plane and around the waist of 2 passive ones out of the plane, as the size of the passive particles varies. Our observed morphology-tailored dynamic transitions, including the influence of the extruded size of the active colloid and the size of the passive colloid, are supported by mesoscale dynamics simulations. Our work reveals a morphology-based strategy for tailoring dynamic states and their transition in assemblies of active and passive colloids, which should be important for steering the movement of active colloids in complex environment [32–35] and designing novel microrobotics [29,36,37] and micromachines [30,38–40].

## Results

### Assemblies of 1 or 2 active colloids with one passive colloid

**Fig. 1.**
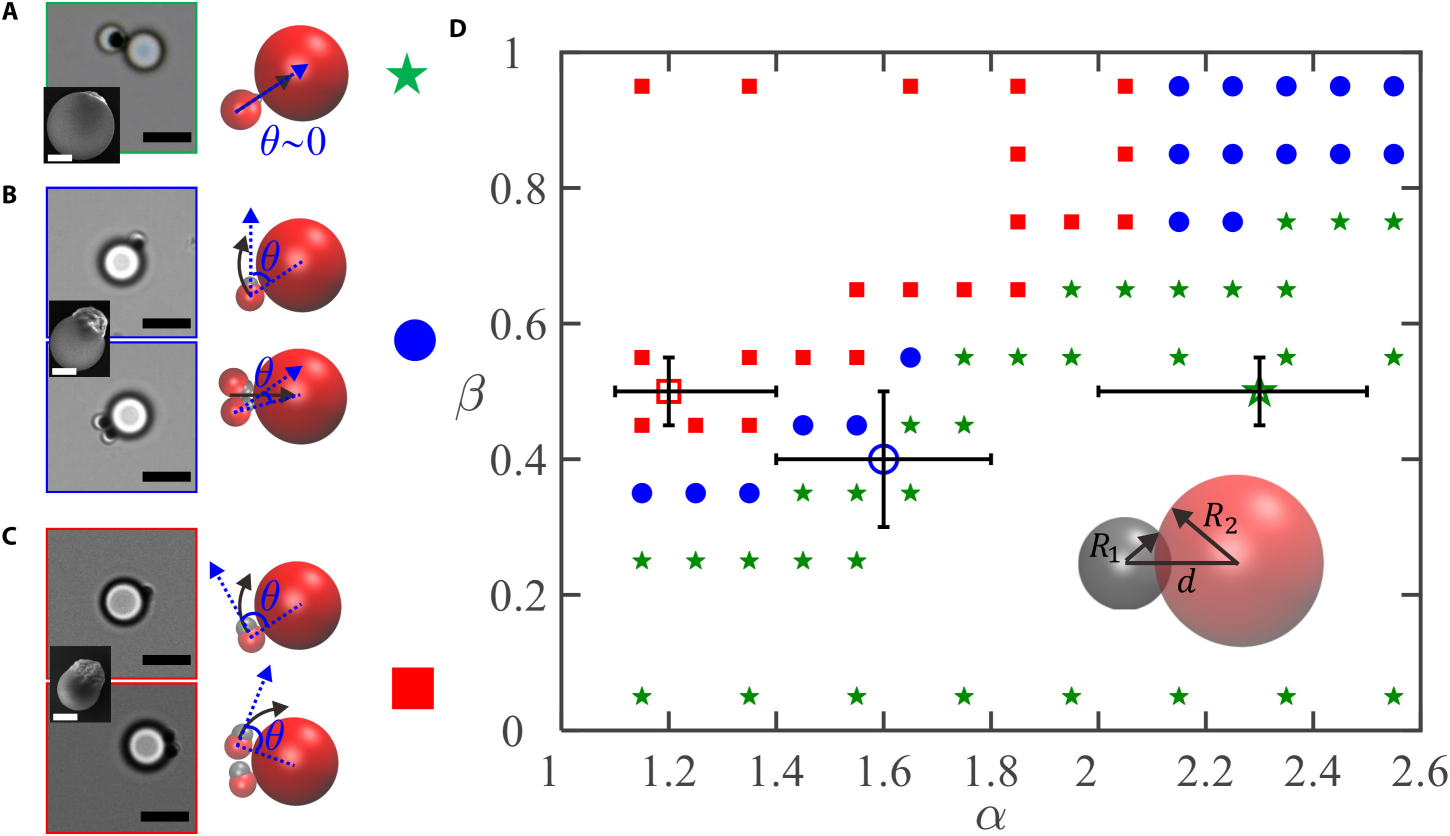
Morphology-tailored transition between translating and orbiting states in the assembly of 1 or 2 active colloids with 1 passive colloid. (A) Active colloid of morphology (2.3, 0.5) exhibits a translating state. (B) Active colloid of morphology (1.6, 0.4) displays an orbiting state for one active colloid and a translating state for 2 active colloids. (C) Active colloid of morphology (1.2, 0.5) shows an orbiting state for both one and 2 active colloids. Schematic illustrations of active assemblies, the tilting angle *θ* and ensuring dynamics are shown on the side. Insets are SEM images of Ag-TPM colloids with different morphologies. (D) Simulated state diagrams of the assembly of active colloids with a passive colloid (filled symbols) together with experimental results (open symbols). The scale bars are 5 and 1 μm in the main figures and the insets, respectively.

The system we employed is a binary mixture of snowman-like active Ag-TPM colloids and larger 3-(trimethoxysilyl) propyl methacrylate (TPM) passive colloids sedimented on a coverslip, which serves as a substrate. The experiments were conducted in the dilute regime to ensure robust and reliable assembly of desired clusters without interference from excessive surrounded particles or reduction of self-propelled speed due to release of ions from high concentration of active colloids. All the experiments were maintained at constant temperature of 22 ^°^C to avoid its complex influence on the thermal noise, solvent viscosity, reaction rate, and diffusivity of ions. Ag-TPM colloids were synthesized following a method we developed earlier [41]. Briefly, hierarchical Ag particles were first synthesized via reducing Ag ions in an aqueous solution by ascorbic acid, which resulted in Ag particles with a diameter of 1.2 ± 0.2 μm. Next, these particles were encapsulated by polymerizable oil, TPM, with the contact angle between the Ag and the oil controlled by a base-triggered dewetting process. Solid Ag-TPM colloids were obtained by ultraviolet (UV)-triggered polymerization; see Fig. 1A to C inset. The morphology of these particles can be described by morphology parameters of $\left(\alpha ,\beta \right)$, where *α* and *β* are the size parameter and the shift parameter, respectively. Here, $\alpha =R_{2}/R_{1}$, and $\beta =d/\left(R_{1}+R_{2}\right)$, with *R*_1_ and *R*_2_ being the radii of the active and passive lobe, respectively, and *d* being the separation between the two (see Fig. 1D inset). Large spherical TPM colloids were synthesized by modifying a procedure described in the literature [42]. We used polymerized TPM particles of different sizes as seed particles, which were fully engulfed in the TPM oil. After the subsequent photopolymerization, we obtained spherical TPM colloids of different sizes. In the presence of hydrogen peroxide (H_2_O_2_), the silver lobe of Ag-TPM colloids can react with H_2_O_2_, creating an asymmetric concentration gradient of Ag^+^, OH^-^ [41] and possibly OOH^-^ ions [43,44]. The diffusivity constants of Ag^+^, OH^-^ and OOH^-^ ions are D_Ag+_ = 1.65×10^-9^ m^2^s^-1^, D_OH-_ = 5.237 × 10^-9^ m^2^s^-1^ and D_OOH-_ = 0.3 × 10^-9^ m^2^s^-1^, respectively. We observe that Ag particles attract nearby negatively charged TPM particles and the self-propulsion of Ag-TPM colloids are led by the Ag part; both indicate a local electric field pointing away from the Ag particle [41]. This suggests that a dominant role of Ag^+^ and OH^-^ ions, as their diffusivity contrast can create an electric field with a direction in agreement with experimental observations. Therefore, we consider Ag-TPM colloid self-propel via self-diffusiophoresis arising from Ag^+^ and OH^-^ ions and interact with passive TPM colloids via diffusiophoresis [41,45] together with excluded volume interaction.

We then investigate the influence of the morphology of the snowman-like active colloids on the active assembly and ensuing dynamics. We find that the tilting angle (*θ*) between the self-propelled direction of the active colloid and the line connecting the centers of the active colloid and the passive colloid, controlled by the morphology of the active colloid, has a significant influence on the dynamics (Fig. 1A to C, and Movie S1). When the exposed area of the active Ag lobe is relatively small and the passive part is large (*α* = 2.3, *β* = 0.5), the self-propelled direction of the active colloid is nearly coincident with the line connecting the active particle and the passive particle, leading to a *θ*~0^°^. As a result, the assembly displays a translating state (Fig. 1A). As the extrusion of the active lobe becomes bigger (*α* = 1.6, *β* = 0.4), both the active part and the passive part of the colloid tend to be in contact with the large passive particle due to phoretic interaction. This results in *θ*~50^°^, such that the active colloid tends to orbit around the large passive colloid (Fig. 1B). Interestingly, when another active colloid joins, the 2 colloids tend to sit side by side, leading to a decrease in *θ* down to ~18^°^. This significantly reduces the orbiting motion and switches the assembly to a translating mode. As the active lobe extrudes even more and the passive part becomes smaller (*α* = 1.2, *β* = 0.5), the active colloid has a tilted angle of ~80^°^, favoring assemblies in the orbiting state (Fig. 1C). We further conducted experiments with the passive TPM colloid replaced by one that has hematite cubed embedded. We clearly observed that the orientation of the passive TPM colloid is nearly unchanged except some small fluctuations (Movie S2), which show that the active colloid is in an orbiting state rather than rotating together with the passive colloid. When a second active colloid joins, the 2 active colloids assemble in a head-to-tail fashion at the perimeter of the passive colloid, causing nearly no change in the tilting angle, and the 2 remain in the orbiting state. Note that the active colloids nearly never come off during the experimental time (a few minutes) once they bind to a passive colloid for all 3 different morphologies (Movie S3), which would correspond to very large (or nearly infinite) binding constants. To facilitate our understanding on this morphology tailored transition between the translating state and the orbiting state, we also conduct computer simulations to explore the dynamic behaviors by systematically tuning the geometric parameters of the active colloid. The state diagram is presented in Fig. 1D, which well captures our experimental observed transition between different dynamic states.

### Assembly of multiple active colloids with one passive colloid

**Fig. 2.**
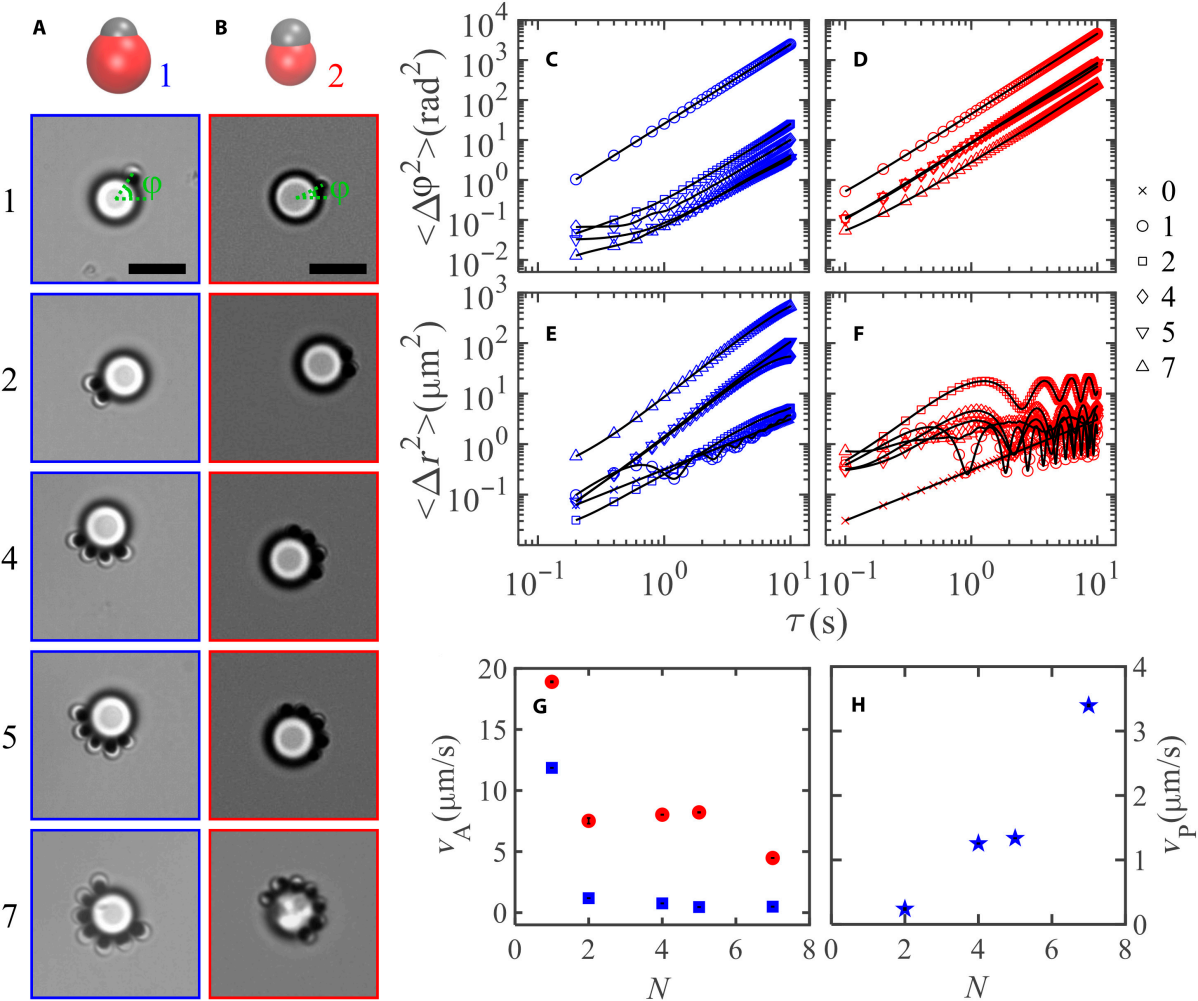
Morphology-dependent translating and orbiting dynamics of assemblies with various number of active colloids. (A and B) Dynamic clusters formed by active colloids of (1.6, 0.4) and (1.2, 0.5), respectively. (C and D) Corresponding MASDs of the attached active colloids. (E and F) Corresponding MSDs of large passive particles. Solid lines are guides to the eye. (G) The linear speed of the active particles relative to the central passive particle. (H) The translational speed of the passive particle for various number of AP1. Scale bars are 5 μm.

We further investigate the dynamics of the assemblies as the number of active colloids changes. As we expect, a similar translating state for both particles with morphology parameter (2.3, 0.5) and (1.6, 0.4) when there are more than one active colloid, in the following we will only focus on assemblies for active colloids of (1.6, 0.4) and (1.2, 0.5), labeled AP1 and AP2, respectively. We first compare the dynamic behavior of their assemblies with one passive colloid of similar size (see Fig. 2A and B and Movie S4). AP1 tends to point toward the center of the large passive colloid (Fig. 2A), i.e. *θ*~0^°^, except for one active particle, while AP2 tends to have a tilting angle close to 90^°^ (Fig. 2B). We define the angle between the central line connecting active and passive particles and the positive X-axis as *φ* (see Fig. 2A and B). The mean angular square displacements (MASDs, 〈Δ*φ*^2^〉) of AP1 decrease significantly when having more than one active particle attached (Fig. 2C), while the MASDs of AP2 were much less affected by the number of active particles (Fig. 2D). We further measured the mean square displacements (MSDs) of the large passive particles in the assemblies. The MSDs of the large passive colloids exhibit clear oscillation with time for one AP1 particle due to the orbiting motion of the active colloid, while increases smoothly with time for 2 or more AP1 particles and become larger with more active particles (Fig. 2E). On the other hand, the MSDs of the large passive colloids are always oscillatory with time, independent on the number of AP2 colloids, displaying little translational motion over time (Fig. 2F). The linear orbiting speeds of AP1 and AP2 were extracted from fitting the MASDs to $r^{2}\langle \Delta \varphi^{2}\rangle =V_{A}^{2}\tau^{2}$ with diffusive term omitted due to their negligible contribution in comparison to the ballistic term, which were shown in Fig. 2G. The speed drops to nearly zero for AP1 as a second or more active particles join in, while after an initial drop in for AP2, the speed essentially levels off with more active colloids. The lack of orbiting dynamics for AP1 makes them better cargo transporters. The transportation speed of the large passive spheres is extracted from fitting their MSDs to a persistent random walk model in the short lag time regime [46], $\langle ∆r^{2}\rangle =4D_{0}\tau +\frac{2}{3}V_{p}^{2}\tau^{2}$, which increases clearly with the number of active colloids, showing their cooperative effect in cargo transportation (Fig. 2H).

**Fig. 3.**
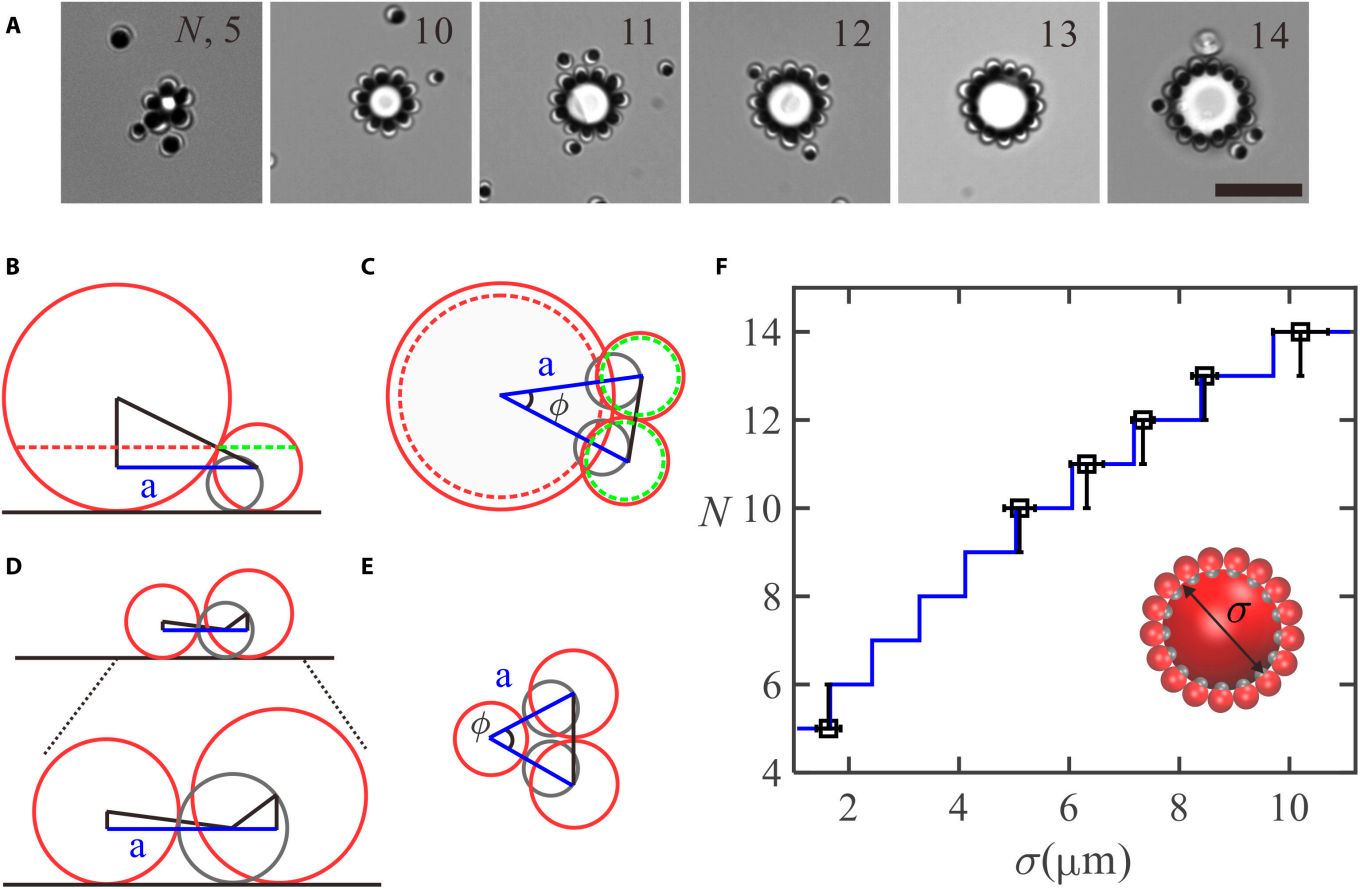
Self-assembly of AP1 around passive particles of various size. (A) Optical images of self-assembled sun-flower-like structures. The scale bar is 10 μm. Schematic side view and top view of 2 closely packed active colloids around one passive colloid of (B and C) critical size *R_c_* and (D and E) below *R_c_*. (F) *N* vs *σ* (symbols: experiments; solid line, Eq. 1). Inset is a schematic on the assembly of a sun-flower.

For AP1, the lack of orbiting dynamics facilitates more and more active colloids to land on the passive colloid, which favors assembly into sun-flower-like structures with full occupancy (Fig. 3A). The coordination number is essentially determined by the size of the passive colloid, which varies from 5 to 14 as the size of passive particle increases from ~1.5 to ~10 μm. To understand the relation between the coordination number and the size of the central passive colloid, we simply consider the geometric constraint on arranging snowman-like particles around a spherical particle. Here, the radius and the diameter of the passive colloid are denoted *R*_3_ and *σ*, respectively. The full occupancy number (*N*) for passive colloid of various size (*σ*) can be determined based on geometric consideration. Fig. 3B and C are schematics on the arrangement when the Ag lobe and the TPM lobe are in contact with the passive sphere simultaneously from which we can calculate the critical size (*R_c_*) of the passive sphere with *R_c_*~1.54 μm. The passive sphere will only touch the passive TPM lobe or the Ag lobe when *R*_3_ > *R_c_* and *R*_3_ < *R_c_* (Fig. 3D and E), respectively. *N* is directly related to the angle *ϕ* formed by 2 adjacent active particles and the passive particle, as sketched in Fig. 3 C via $N=\left\lfloor 2\pi /\phi \right\rfloor$, where $\phi =\text{acos}\left(1-2R_{2}^{2}/a^{2}\right)$. We can then express *N* as a function of *σ* (for details, see Supplementary Materials),

N=2π/acos1−2R22/2R1σ+β2R2+R12−R2−R122,σ<2Rc2π/acos1−R2/σ,σ≥2Rc (1)

Eq. 1 was plotted in Fig. 3F, which can well capture the experimentally meansured *N* as a function of the diameter *σ* of the passive colloid.

**Fig. 4.**
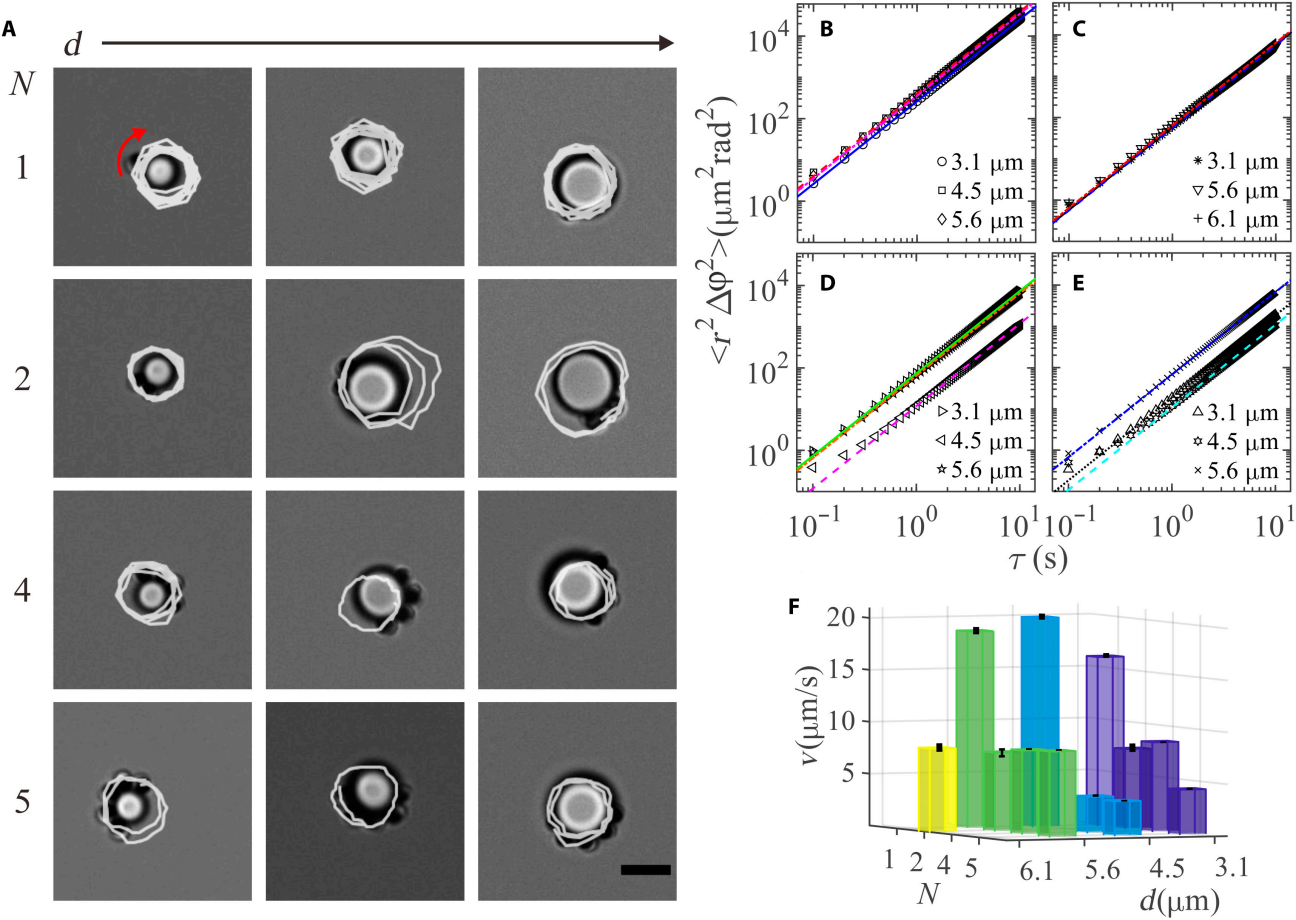
Orbiting dynamics for assemblies of AP2 with one passive colloid as the number of active colloids and the size of the passive colloid vary. (A) Optical images of various assemblies with overlaid trajectories of active colloids over 5 seconds (the white lines); the red curved arrow indicates the orbiting direction; (B to E) The MASDs for 1, 2, 4, and 5 active colloids; lines are fit to *r*^2^〈∆*φ*^2^〉 = *r*^2^*ω*^2^*τ*^2^ = *v*^2^*τ*^2^ with diffusive term ignored. (F) Linear speed *v* of the active particles obtained from the fits. Scale bars are 5 μm.

We then focus on the orbiting dynamics of the assemblies arising from AP2 and passive colloids (Movie S5). We first investigate the influence of the size of the passive particles and the number of AP2 (Fig. 4A). For 1 and 2 AP2, the orbiting speed are essentially independent on the size of the passive particles (Fig. 4B and C), similar to a recent report on the speed of Pt-based active colloids around fixed posts [25]. As more active particles join, they tend to be bonded closely to each other due to diffusiophoretic interactions and orbit together around the passive colloid (Fig. 4D and E). The corresponding propulsion linear speed of active colloids was summarized in Fig. 4F for various size of the passive colloid and various number of active colloids. Here, we observe a slowing down in the speed of active colloids when a second one joins instead of cooperative, synergistic swimming (i.e. increase of speed) as observed in an early report on Pt-based Janus active colloids [25]. In their case, the active colloids are well separately from each other due to a short-range repulsion arising from each other’s pusher-like flow field, with more efficient swimming attributed to hydrodynamics. In our case, active colloids are tightly bounded together. They can largely be considered as one object, which is less broken in symmetry and therefore will have a reduced swimming speed.

Furthermore, the active colloids may not be well aligned, which influence the speed together with the polydispersity of particle size and morphology, making it difficult to draw a conclusion on the speed dependence on the size of passive particles.

In addition, we studied the motion of the passive colloids, with their MSDs shown in Fig. S1. Passive colloids exhibit clear oscillatory motion, whose frequency can be obtained by examining the duration between 2 neighboring valleys in the MSDs. The oscillation frequencies for various sizes of passive colloids and various number of active colloids are presented in Table S1. For one active colloid, there is a clear decrease in the frequency as the size of the passive colloid increases, which is largely due to the increased time for the active colloid to complete one round. For the same passive colloid, the oscillation frequency drops for more than one active colloids in comparison to one, which can be ascribed to a decrease in the self-propelled speed of active colloids.

### Assemblies of 1 active colloid and 2 passive colloids

**Fig. 5.**
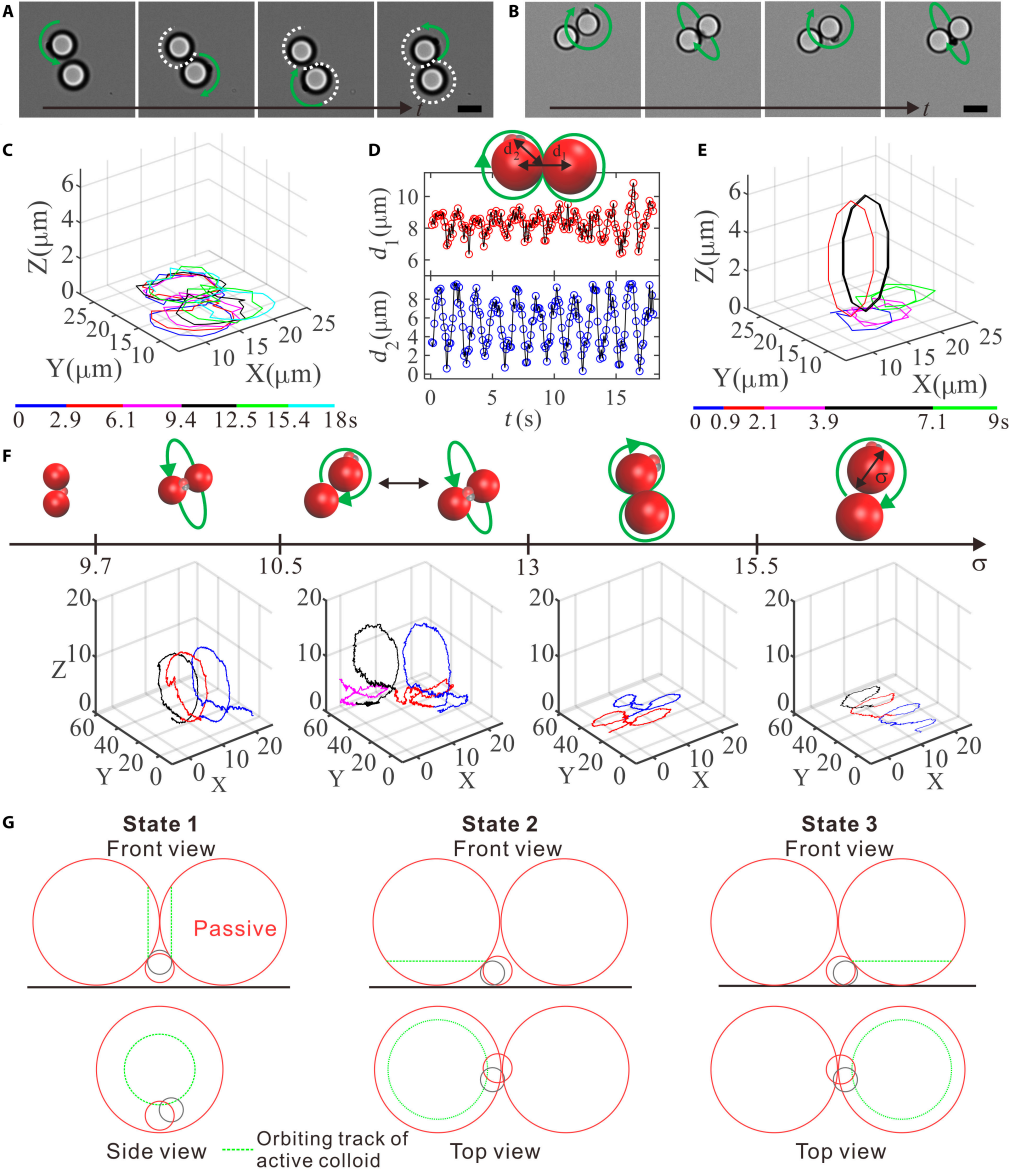
Steering dynamics of a AP2 around 2 passive colloids. (A) Optical images of “8”-like steering dynamics (*σ*~8.1 μm) and (B) hopping dynamics between circling states around one passive particle in the plane and around the waist of 2 passive ones out of the plane (*σ*~6.6 μm). The scale bars are 5 μm. (C) The corresponding trajectory of the AP2 in (A). (D) The distance *d*_1_ of the 2 large spheres and the distance *d*_2_ between the active particle and the midpoint of the 2 large spheres vs time. (E) The trajectory of the AP2 in (B). (F) Simulated state diagram and corresponding typical trajectories. (G) Three orbiting states of AP2.

In the end, we investigate the orbiting dynamics for the assemblies of one AP2 and 2 large passive spheres. The concentration gradient created locally by the active colloid not only leads to self-propulsion but also its affinity to nearby objects or surfaces. This in fact is the reason why an active colloid orbiting around a passive colloid close to the substrate. Meanwhile, the chemical gradient follows the motion of the active colloid, which can also attract another nearby passive particle. This can lead to an assembly of 1 active colloid and 2 passive colloids via phoretic attraction. Interestingly, we observed different dynamic states even when the size of the passive particle changes slightly from 8.1 μm (Fig. 5A and Movie S6) to 6.6 μm (Fig. 5B and Movie S7). In the first case, we find that the active colloid circles around the 2 passive particles with repeating “8”-like trajectories (Fig. 5C). This type of motion is particularly important for the design of weaving micromachines[30]. We find that the separation between the 2 passive spheres (d_1_) is strongly correlated to the distance of the active colloid to the midpoint connecting the 2 passive spheres (d_2_), see Fig. 5D, and as a result the passive particles display a to and fro motion in response to this gradient. When d_2_ increases, the active colloid tends to pull the passive colloid attached to it away from the other one, leading to an increase in d_1_. As d_2_ decreases, the active colloid pulls the passive colloid attached to it toward the other one. This leads to a synchrony between the variation of d_1_ and d_2_. With a slight decrease in the size of the passive particles, we find that the active particle now switches back and forth between 2 modes of motion: revolving around one passive colloid and revolving around the waist of the 2 joining passive particles. The active particle orbits around a passive particle for several rounds before climbing up against gravity and circling around the waist of the 2 passive particles. After circling for a few rounds, the active colloid can transit back to the orbiting state around one passive particle. This process can be repeated for several times, as shown in the trajectories in Fig. 5E. In fact, more than 2 passive colloids can be assembled with one active colloid, which can further enrich the dynamics. One example is given for an assembly of 1 active particle and 3 passive ones (see Movie S8). The active one orbits around one passive particle, leading to consistent rotation of the entire cluster. However, dynamics of this kind is not beyond the basic modes of motion we discussed above, which therefore will not be pursued further.

To understand the underlying reason for the dynamic transition we observed, we carry out mesoscale fluid dynamics simulations, with results summarized in Fig. 5F (see also Movie S9). When the diameter (*σ*) of the passive colloidal particles is below 9.6, the geometric threshold for passing through the gap between 2 touched passive particles and the substrate, the active colloid will simply be stuck between 2 passive colloids without orbiting. As *σ* is beyond this limit but less than a certain threshold, ~10.5, the active particle shows preferred circular motion around the axle created by the joint of the 2 passive particles. When the value of *σ* gradually increases, the active colloid can hop between 2 dynamic bistable states of revolving around one passive colloid and revolving around the axle of the 2 passive colloids. When *σ* further increases, we observe that the active colloid exhibits “8”-like path around the 2 passive colloids close to the substrate. Finally, if *σ* further increases, the active colloid can only orbit around one passive colloid on the substrate.

Combining experiments with computer simulations, the observed dynamic behaviors can be qualitatively understood as the following. Due to its strong affinity to nearby surfaces arising from phoresis and hydrodynamics [24,25,47,48], the active colloid in an assembly of 1 active and 2 equal-size passive colloids have 3 preferred orbiting tracks or dynamic states (see Fig. 5G): one is around the axle created by the joint of the 2 passive particles (state 1), and the other two are surrounding one passive colloid close to the substrate (state 2 and 3), which are identical. For passive particles of size ~6.6 μm, the states 1 to 3 are equistable to each other. When an active colloid is in state 2 (or 3), its active lobe can be close enough to the surface of the second passive colloid (~0.7 μm) such that the active colloid feels a strong attractive force from it, which allows the active colloid hopping to state 1 against gravity (a height of ~0.3 μm is estimated, which corresponds to a gravitation energy of ~6*k_B_T*). The delicate balance between the affinity to the second passive colloid and the gravitational energy penalty allows the active colloid hopping back and forth. As the size of passive colloids increased to ~8.1 μm, the distance from the surface of the active lobe to that of the other passive colloid is nearly doubled (~1.4 μm) and the distance to lift the active colloid from the substrate is nearly tripled (~0.8 μm, corresponding to gravitational energy of ~17*k_B_T*). As a result, the active colloid can no longer hop from state 2 (or 3) to state 1. Instead, a transition between state 2 and 3 is preferred, resulting 8-like motion. This cyclic 8-like motion has been proposed earlier for an active dimer moving around and being bound to 2 fixed circular posts by depletion interaction [30]. Note that we realized in experiments this novel dynamic state using chemically driven active colloids with a self-created concentration gradient of solutes that are responsible for both self-propulsion and attraction to nearly boundaries. As the size of the passive colloids further increases, it is readily conceivable that the active colloid can hardly feel the presence of the other one due to their large separation, which will then leave the active colloid orbiting around one passive colloid near the substrate. These dynamic transitions were well supported by our computer simulations.

## Discussion

To summarize, we have studied experimentally and numerically the dynamic state transitions in mixtures of active and passive colloids at constant temperature in the dilute regime. We reveal that gradually increasing the extrusion of active part of the active colloid leads to transition from translational motion for one active particle, orbiting motion for one active particle and translational motion for 2 active particles, to orbiting motion for one or more active particles. This morphology-controlled dynamic behaviors of assemblies of a couple of active colloids with a passive colloid are well captured by the simulated dynamic state diagram. We show that active colloids with smaller extruded active part can work together as large cargo transporters and serve as building blocks for constructing “sunflower”-like assemblies while those with larger extruded active part display orbiting train-like dynamics with essentially size-independent linear speed for assemblies with only one passive colloid. We further observed intriguing dynamic states in assemblies of one active colloid with 2 passive colloids, including persistent “8”-like steering motion, which is central to the design of microscopic weaving machine [30], and hopping between metastable circling states around one passive particle in the plane and around the waist of 2 passive ones out of the plane. Combining with computer simulations, we reveal that the dynamic transition is controlled by the size of the passive colloids, which tunes the competition among metastable states of circling around different passive particles on the plane and axle of the 2 passive particles out of the plane. Our work reveals principles of tailoring the dynamic states of active and passive colloidal assemblies via the morphology of active colloids and the size of passive colloids, which are important for designing active functional materials of various applications, such as micro-machines, cargo delivery, and steering systems.

## Materials and Methods

### Computer simulations

Three-dimensional mesoscale fluid simulations were conducted to investigate the dynamics and assemblies of active and passive colloidal mixtures. The method properly takes hydrodynamics, mass transport, thermal fluctuations and diffusiophoretic effect into consideration. In the simulations, the solvent molecules were modeled as point-like particles using multiparticle collision dynamics, while the colloid–colloid and colloid–molecule interactions were described by standard molecular dynamics. The Ag part of active colloid was modeled as a catalytic sphere (shown as gray) of radius *R*_1_ = 2 (in simulation units) embedded in a passive sphere (shown as red) of *R*_2_ = 2*α*, with its center position determined by the value of *β*; see Fig. S2. The larger passive colloids were modeled as a simple sphere. To simulate the catalytic reaction, reactant particles were turned into product particles close to the catalytic part, while reactant particles were continuously input at regions far away from the active part via the inverse reaction. The reactant and the product particles interact with all the colloids via a repulsive or an attractive interaction [49,50] of the from of *U_A_* and *U_B_* (see Supplementary Materials), respectively. This leads to self-propulsion (self-diffusiophoresis) of the active colloid in the direction of its catalytic part and attractive diffusiophoretic interaction with passive colloids, in agreement with experimental observation. Furthermore, a boundary wall in the Z direction that exerts repulsive interactions on the active and passive colloids is implemented to simulate the substrate, while periodic boundary conditions are implemented in both X and Y directions. The fluid particles couple with the walls via the bounce-back collision rule to realize a nonslip boundary condition. All colloidal particles in the simulations also tend to stay near the substrate due to a small external force applied in the -Z direction, mimicking the residual gravity in the experiments. In the simulation, a proper temperature is chosen to reproduce experimentally observed phenomena. Higher temperature can give rise to larger thermal fluctuations, which weaken the binding between active and passive colloids and make clusters unstable, while at lower temperature thermal fluctuations are expected to be overwhelmed by the attraction between the active and passive colloids, thus making it more difficult for the active colloid hopping between different dynamic states. More details on the simulations were provided in the Supplementary Materials [51].

### Chemicals

The following chemicals were supplied by Sigma-Aldrich: Silver nitrate (ACS reagent ≥ 99%), hydrogen peroxide (30% wt.), ethanol, L-ascorbic acid (ACS reagent ≥ 99%), 3-trimethoxysilyl propyl methacrylate (TPM, ≥ 98%), 1-hydroxycyclohexyl phenyl ketone (HCPK), and isopropyl alcohol. Ammonia solution (25% wt.) was obtained from Acros Organics. Deionized water (DI; Millipore, 18.2 mΩ) was used in all experiments.

### Synthesis of Ag-TPM colloids

Snowman-like Ag-TPM Janus colloids were synthesized following a method we reported earlier [39]. First, Ag seed particles were synthesized. Briefly, 50 mg of ascorbic acid was dissolved in 10 ml of DI water under magnetic stirring for 5 min in a 30-ml glass vial. Then, 0.2 ml of 0.5 M AgNO_3_ solution was added into the vial. The synthesis reaction was carried out for 5 min. The as-synthesized particles were sonicated for 5 min and washed 3 times with a 1:1 mixture of ethanol and DI water. The particles were finally dispersed in 5 ml of DI water. Then, we start to synthesize Ag-TPM Janus colloids. Briefly, TPM oil was first hydrolyzed in DI water under magnetic stirring in a 1:10 V/V ratio to form hydrolyzed TPM (hTPM). Next, 13 ml of DI water and 3.5 μl of ammonia solution were added to a 30-ml glass vial before adding in 0.2 ml of the Ag particles suspension. After sonicating for 1 min, 1.0 ml of hTPM was added with the mixture gently shaken by hand. The mixture was left undisturbed for 30 min. Then, HCPK was added and the mixture were illuminated under UV light (365 nm, 5.6 mW/cm^2^, Spectroline XX-15A benchtop UV lamp) for 10 min for photopolymerization. The resulted particles were washed twice with ethanol and 3 times with DI water and then suspended in 5 ml of DI water.

### Synthesis of TPM passive spheres

First, 13.0 ml of DI water and 3.5 μl of the ammonia solution were mixed in a 30-ml glass vial, followed by the addition of 1.0 ml of hTPM. The mixture was gently shaken with hand, which was left undisturbed for 40 min. Then, 50 mg of HCPK was added and the mixture was under UV illumination for 20 min to initiate photo-polymerization. The resulted TPM particles were collected by centrifugation at 1,500 rpm for 10 min, washed twice with ethanol and 3 times with DI water, and then suspended in 10 ml of DI water. Larger TPM particles were synthesized by mixing 1 ml of the above TPM particle suspension with 9 ml of DI water and 3.5 μl of ammonia solution, followed by addition of 4 ml of hTPM and subsequent photopolymerization. Even larger TPM particles can be obtained by further seeded growth.

### Characterization

Optical images were acquired with an Olympus IX73 inverted microscope equipped with a 60× oil immersion objective lens (PLAPON; NA = 1.42) and a xiQ digital camera (Ximea) or a Prime-BSI camera (Photometrics). Scanning electron microscopy (SEM) images were acquired on a FEI-Scios field emission scanning electron microscope (FEI-SEM) operated at 5 kV.

## Data Availability

All data are presented in the paper and the Supplementary Materials.

## Supplementary Materials

Supplementary 1 Sections S1 to S3 Figs. S1 to S2 Movies S1 to S9 Table S1
