# Supplementary material for: Morphology-Tailored Dynamic State Transition in Active-Passive Colloidal Assemblies
Source: Research (Wash D C). 2024 Jan 24;7:0304. doi: 10.34133/research.0304 (PMC10807723; doi:10.34133/research.0304)
Supplement: Supplementary 1 — Sections S1 to S3 Figs. S1 to S2 Movies S1 to S9 Table S1 [file research.0304.f1.zip › Supplemental Material_Clean Version.docx]

Supplemental Material

**Morphology-tailored** **dynamic state transition in active-passive** **colloidal assemblies**

Nan Yu, Zameer Hassan Shah, Mingcheng Yang^#^, and Yongxiang Gao^*^

Nan Yu

Institute for Advanced Study, Shenzhen University, 518060, Shenzhen, China

Key Laboratory of Optoelectronic Device and Systems of Ministry of Education and Guangdong Province

College of Optoelectronic Engineering, Shenzhen University, 518060, Shenzhen, China

Zameer Hassan Shah

Institute for Advanced Study, Shenzhen University, 518060, Shenzhen, China

Key Laboratory of Optoelectronic Device and Systems of Ministry of Education and Guangdong Province

College of Optoelectronic Engineering, Shenzhen University, 518060, Shenzhen, China

M. Yang

Beijing National Laboratory for Condensed Matter Physics and Laboratory of Soft Matter Physics, Institute

of Physics, Chinese Academy of Sciences, Beijing 100190, China

School of Physical Sciences, University of Chinese Academy of Sciences, Beijing 100049, China

Songshan Lake Materials Laboratory, Dongguan, Guangdong 523808, China

mcyang@iphy.ac.cn

Y. Gao

Institute for Advanced Study, Shenzhen University, 518060, Shenzhen, China

yongxiang.gao@szu.edu.cn

**1. Oscillations of passive colloids as active colloids orbiting**


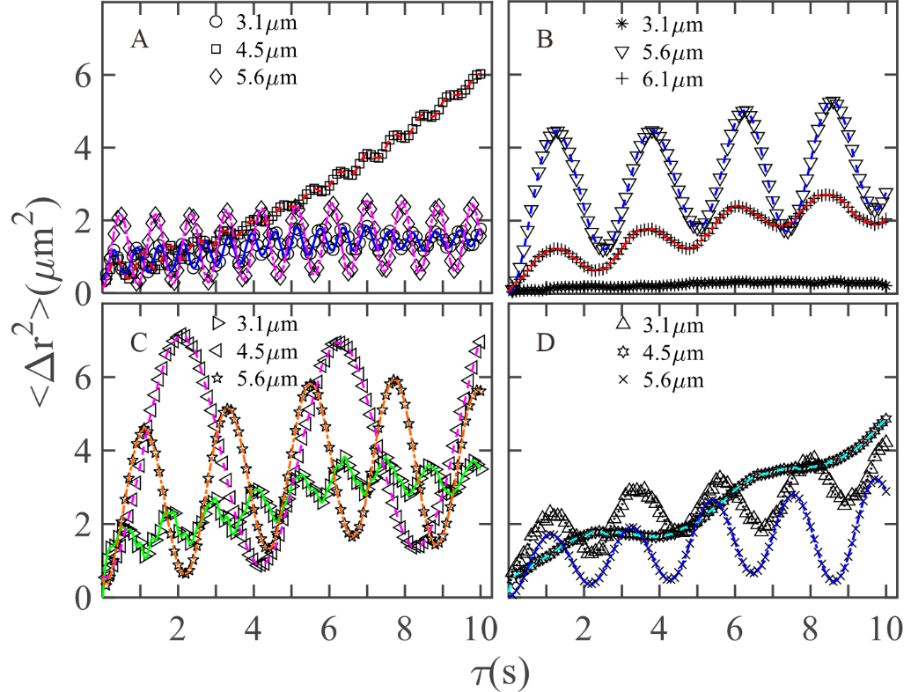


Figure S1 The MSDs of passive colloids as the number of active colloids varies. (A)-(D) corresponding to 1, 2, 4 and 5 active colloids respectively. The MSD value is a quarter of the true value for passive colloid of $5.6 \mu m$shown in (B) for clarity.

Table S1. The oscillatory frequencies (f, Hz) of passive colloids for different number of active colloids and different size of passive colloids.

| $N$ $D\left( \mu m \right)$ | 1 | 2 | 3 | 4 | 5 |
| --- | --- | --- | --- | --- | --- |
| 3.1 | 1.66±0.12 | NA | NA | 0.87±0.04 | 0.45±0.04 |
| 4.5 | 1.39±0.08 | NA | 0.26±0.01 | 0.25±0.01 | 0.24±0.01 |
| 5.6 | 1.08±0.05 | 0.41±0.02 | NA | 0.45±0.02 | 0.47±0.01 |
| 6.1 | NA | 0.42±0.03 | NA | NA | NA |

**2. Geometric consideration on the occupancy number *N***

When $R_{3}>R_{c}$, the distance $a$ shown in Figure 3B and C can be determined via

$a=\sqrt{\left( R_{3}+R_{2} \right)^{2}-\left( R_{3}-R_{2} \right)^{2}}=2\sqrt{{R_{2}R}_{3}}$ (S1)

When $R_{3}<R_{c}$ (Figure 3D and E), the passive colloid can only be in contact with the Ag lobe such that the distance $a$ can be solved via the following equation,

$a=\sqrt{\left( R_{3}+R_{1} \right)^{2}-\left( R_{3}-R_{1} \right)^{2}}+\sqrt{{\beta^{2}\left( R_{2}+R_{1} \right)}^{2}-\left( R_{2}-R_{1} \right)^{2}}$. (S2)

The critical size $R_{c}$ is determined by setting the above two equations equal, from which we can solve $R_{c}=1.54 \mu m$. The angle ($\phi$) formed between two touched active colloids with the passive colloid can be calculated by the following equation,

$\phi=acos\left[ \left( a^{2}+a^{2}-\left( 2R_{2} \right)^{2} \right)/{{2a}^{2}} \right]=\mathrm{acos} [ \left( a^{2}-2R_{2}^{2} \right)/a^{2}]$ (S3)

which can be used to determine the full occupancy number ($N$) via $N=\left\lfloor2\pi/\phi\right\rfloor$. We can therefore express $N$ as a function of the diameter $\sigma$ of the passive colloid as Eq 1 in the main text.

**3. Computer simulations**

In mesoscale fluid simulations, we use coarse-grained method to model the solvent as a large number $N$ of point-like particles of mass $m$ with continuous positions $r_{i}(t)$ and velocities $v_{i}(t)$. The algorithm consists of alternating streaming and collision steps. In the streaming step, all the solvent particles move ballistically for a time $h$. In the collision step, particles are sorted into a cube lattice with lattice size $a$, and interchange momentum relative to the center-of-mass velocity of each collision cell. This collision rule not only locally conserves mass, linear momentum, energy, and angular momentum, but also properly captures hydrodynamic interactions, mass diffusion, thermal fluctuations, and thermal conduction. Simulation units are reduced by setting $a=1$, $m=1$, and the system mean temperature is set to be $k_{B}\bar{T}=0.5$, with $k_{B}$ being the Boltzmann constant. From the basic MPC units, the time unit is $a\sqrt{m/{k_{B}\bar{T}}}$. We employ MPC parameters $h=0.1$ and the mean number of solvent particles per cell $\rho=10$. In our simulations the stochastic rotation collision rule with fixed collision angle $\alpha={2\pi}/3$ is employed. According to the above parameters, we can approximately calculate the Schmidt number as $S_{C}\simeq25.2$, which corresponds to liquid-like dynamics.

To mimic the catalytic reaction on the catalytic part of the active particle, we consider the solvent contains two species A (reactant) and B (product). When species A approaches to the catalytic part, it will turn into species B with a reaction probability $p=1$; while inverse reaction far away from the colloids is implemented in order to input the fuel, with a reaction probability $p=0.001$. Solvent species A and B interact with the colloids through different potentials, as displayed in **Figure S2**. The interactions of species A with the active colloid and the passive colloid are both described by a truncated and shifted Lennard-Jones potential, $U_{A}\left( r \right)=4\varepsilon\left[ \left( R/r \right)^{24}-\left( R/r \right)^{12} \right]+\varepsilon$, for$r\leq r_{c}$, but with different particle radius $R$. Here, $r$ is the distance from the colloid center to the solvent molecules, $\varepsilon=1$ is the potential intensity, and $r_{c}$ is the corresponding interaction range. While, solvent species B interacts with the passive colloid and the active particle via $U_{B}\left( r \right)$, which is constructed from the repulsive Lennard-Jones potential $U_{A}\left( r \right)$ using a cubic spline interpolation, producing an attractive tail. The colloidal particles and their neighbouring solvent evolve according to molecular dynamics scheme, in which the particle equations of motion are integrated by the velocity-Verlet algorithm with a time step $\Delta t=h/50$.

**
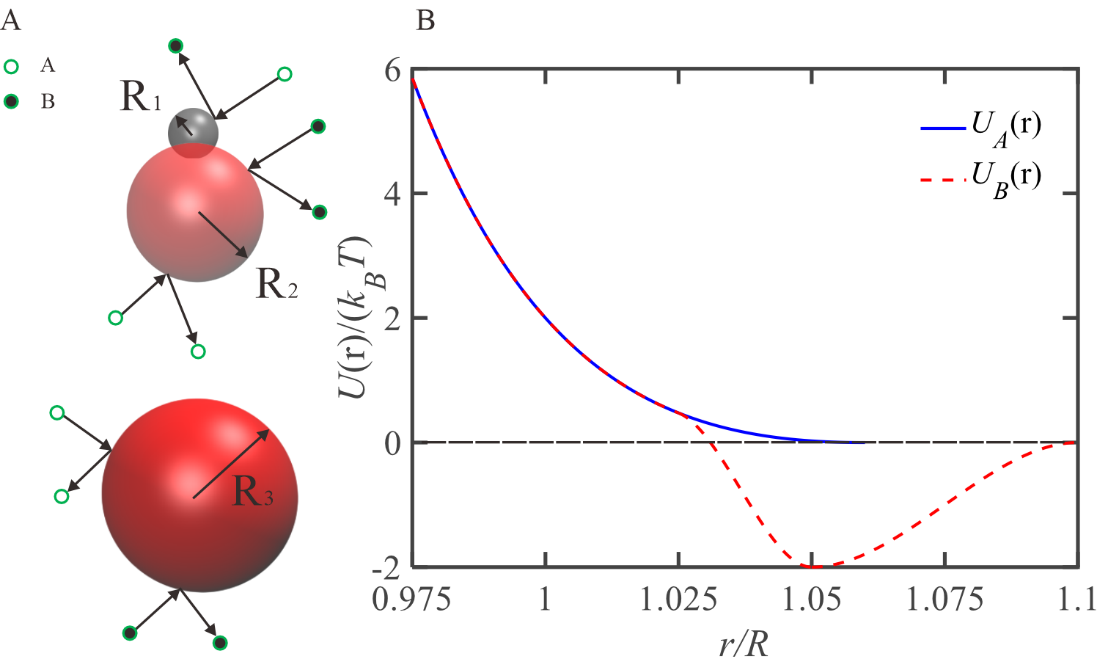
**

**Figure S2.** A) Schematic representation of the catalytic (gray sphere) and non-catalytic (red sphere) part of the snowman-like active colloid and the bigger passive sphere colloid. The chemical reaction A→B happens on the surface of the gray sphere. B) Potential interactions of spheres and solvent species A ($U_{A}(r)$ blue solid line) and species B ($U_{B}(r)$ red dashed line). Here, $U_{A}(r)$ is purely repulsive, while $U_{B}(r)$ has an attractive tail. $R$ can represent $R_{1}$, $R_{2}$ and $R_{3}$.

**4. Supplemental Videos**

**Supplemental Video S1:** Dynamic behaviors for one and two active colloids of morphology (2.3, 0.5), (1.6, 0.4) and (1.2, 0.5) with one passive colloid.

**Supplemental Video S2:** Orbiting of one active colloid of morphology (1.6, 0.4) around one passive colloid with hematite cubed embedded.

**Supplemental Video S3:** Binding processes of active colloids of morphology (2.3, 0.5), (1.6, 0.4) and (1.2, 0.5).

**Supplemental Video S4:** Translating and orbiting dynamics AP1 and AP2 as the number of active colloids vary.

**Supplemental Video S5:** Orbiting dynamics of AP2 as the number of active colloids and the size of passive colloid vary.

**Supplemental Video S6:** Persistent ‘8’-like cyclic motion for one AP2 and two passive colloids.

**Supplemental Video S7:** Dynamic transition between circling states around one passive particle in the plane and around the waist of two passive ones out of the plane for one AP2 and two passive colloids.

**Supplemental Video S8:** Dynamics of an assembly of one AP2 and three passive colloids.

**Supplemental Video S9:** Simulated dynamic states for one active colloid and two passive colloids as the radii of the passive colloids vary.

Scalebars are 5 µm in all the videos.
